# Supplementary material for: The Small RNA Universe of Capitella teleta
Source: Front Mol Biosci. 2022 Feb 25;9:802814. doi: 10.3389/fmolb.2022.802814 (PMC8915122; doi:10.3389/fmolb.2022.802814)
Supplement: Supplementary file 1 [file DataSheet1.ZIP › Supplement/known/CAPTEscaffold_174_10477.pdf]

Provisional ID	: CAPTEscaffold_174_10477
Score total	: 575916.7
Score for star read(s)	: 3.9
Score for read counts	: 575906.7
Score for mfe	: 1.4
Score for randfold	: 1.6
Score for cons. seed	: 3
Total read count	: 1129628
Mature read count	: 1129382
Loop read count	: 0
Star read count	: 246

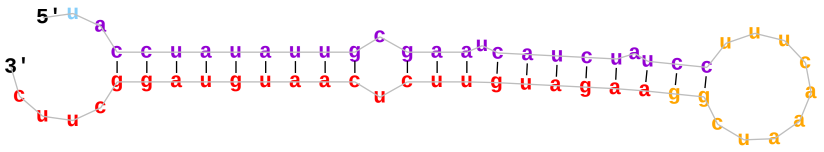

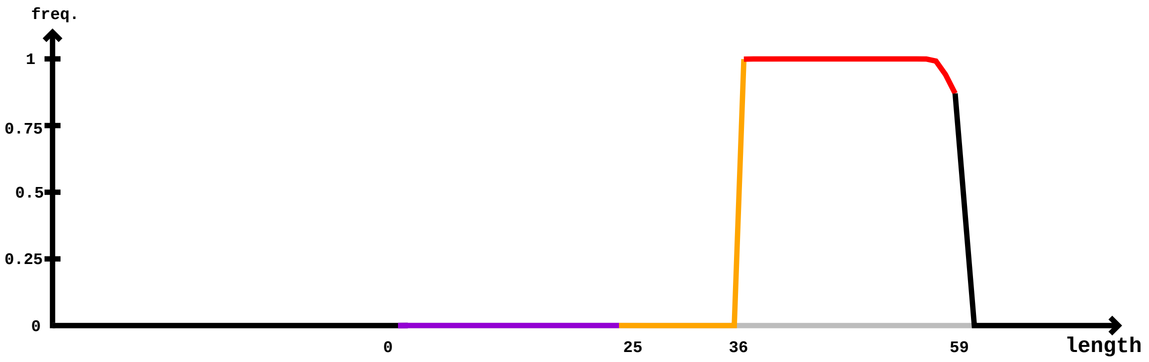

Star **Mature**

5'- gaucaccauggcgacggaccuugaccgauuugaguaccuauauugcgaaucaucuauccuuucaaaucggaagauguucucaauguaggcuucuuaagucgcgaucaau -3'	obs		
gaucaccauggcgacggaccuugaccgauuugaguaccuauauugcgaaucaucuauccuuucaaaucggaagauguucucaauguaggcuucuuaagucgcgaucaau	exp		
gaucaccauggcgacggaccuugaccgauuugaguaccuauauugcgaaucaucuauccuuucaaaucggaagauguucucaauguaggcuucuuaagucgcgaucaau	known		
((((..((.(((....).)).))..(((((((((..(((((((((.(((.(((((.(((.........))))))))))).)))))))))...))))))))).)))).. .	reads	mm	sample
................................aguaccuauauugcgaaucaucu..	1	0	seq
..................................uaccuauauugcgaaucaucuauc...	2	0	seq
...................................accuauauugcgaaucaucuau..	5	0	seq
...................................accuauauugcgaaucaucuauc...	70	0	seq
...................................accuauauugcAaaucaucuaucc..	1	1	seq
...................................accuauauugcgaaucaucuaucU..	23	1	seq
...................................accAauauugcgaaucaucuaucc..	1	1	seq
...................................Gccuauauugcgaaucaucuaucc..	1	1	seq
...................................accuauauugcgaaucaucuaucc..	141	0	seq
...................................accuauauugcgaaucaucuauccu...	1	0	seq
....aucggaagauguucucaauguaggcuuc................	1	0	seq
....Caagauguucucaauguaggcuu.................	6	1	seq
....Caagauguucucaauguaggcuuc................	133	1	seq
....Aaagauguucucaauguaggcuuc................	1	1	seq
....gaagauguucucaauguaggcuuc................	2	0	seq
....aagauguucucaauguag.....................	21	0	seq
....Nagauguucucaauguagg....................	1	1	seq
....aagauguuUucaauguagg....................	1	1	seq
....aagauguucucaauguagA....................	8	1	seq
....aagauguAcucaauguagg....................	1	1	seq
....Gagauguucucaauguagg....................	1	1	seq
....aagauguucucaauguagg....................	326	0	seq
....aagauguucucaauguaAgc...................	3	1	seq
....aagauguucCcaauguaggc...................	1	1	seq
....aagauguucucaauguagAc...................	1	1	seq
....aagauguucucaauguaggA...................	797	1	seq
....aagauguuAucaauguaggc...................	2	1	seq
....aagaugGucucaauguaggc...................	1	1	seq
....aagauguucucaaugAaggc...................	4	1	seq
....Uagauguucucaauguaggc...................	2	1	seq
....aagauguucucaaugNaggc...................	1	1	seq
....aagauAuucucaauguaggc...................	4	1	seq

```
                                   Star                   Mature
gaucaccauggcgacggaccuugaccgauuugaguaccuauauugcgaaucaucuauccuuucaaaucggaagauguucucaauguaggcuucuuaagucgcgaucaau
......................................................................aagaAguucucaauguaggc................... 6     1     seq
......................................................................aagauguucAcaauguaggc................... 1     1     seq
......................................................................aagauguucucaauguaggG................... 1     1     seq
......................................................................aagauguucuAaauguaggc................... 2     1     seq
......................................................................aagauguucucaauguaggc................... 7777  0     seq
......................................................................aagauguucucaauguaggU................... 6     1     seq
......................................................................aagauNuucucaauguaggc................... 1     1     seq
......................................................................aagaCguucucaauguaggc................... 1     1     seq
......................................................................aagauguucucaauguagCc................... 1     1     seq
......................................................................aagauCuucucaauguaggc................... 2     1     seq
......................................................................aagauguucucaauguGggc................... 2     1     seq
......................................................................aagauguucucaauguagUc................... 2     1     seq
......................................................................aagaugAucucaauguaggc................... 7     1     seq
......................................................................aagauguucucaGuguaggc................... 1     1     seq
......................................................................aagauguucucUauguaggc................... 1     1     seq
......................................................................aagauguucucaUuguaggc................... 3     1     seq
......................................................................aagauguCcucaauguaggc................... 2     1     seq
......................................................................Nagauguucucaauguaggc................... 1     1     seq
......................................................................aagauguucucaauguUggc................... 1     1     seq
......................................................................Gagauguucucaauguaggc................... 1     1     seq
......................................................................aagauguucucGauguaggc................... 2     1     seq
......................................................................aagauguucucaauguaggUu.................. 8     1     seq
......................................................................aagauguucuGaauguaggcu.................. 6     1     seq
......................................................................aagauguucucaauguaggcA.................. 41    1     seq
......................................................................aagauguucucaauguaUgcu.................. 1     1     seq
......................................................................aagauguucucUauguaggcu.................. 7     1     seq
......................................................................aagaugGucucaauguaggcu.................. 9     1     seq
......................................................................aagauNuucucaauguaggcu.................. 1     1     seq
......................................................................aagauguucucaaugGaggcu.................. 5     1     seq
......................................................................aaUauguucucaauguaggcu.................. 4     1     seq
......................................................................aaAauguucucaauguaggcu.................. 8     1     seq
......................................................................aagauguuAucaauguaggcu.................. 9     1     seq
......................................................................aagauguucucaauguGggcu.................. 7     1     seq
......................................................................aagauguucucaaAguaggcu.................. 16    1     seq
......................................................................aagauguuGucaauguaggcu.................. 5     1     seq
......................................................................aagauguucucaauguaggAu.................. 16    1     seq
......................................................................aagaugAucucaauguaggcu.................. 49    1     seq
......................................................................aagauCuucucaauguaggcu.................. 5     1     seq
......................................................................aagauguAcucaauguaggcu.................. 17    1     seq
......................................................................aagauAuucucaauguaggcu.................. 6     1     seq
......................................................................aagGuguucucaauguaggcu.................. 4     1     seq
......................................................................aagauguucucaaCguaggcu.................. 4     1     seq
......................................................................aaCauguucucaauguaggcu.................. 2     1     seq
......................................................................Gagauguucucaauguaggcu.................. 13    1     seq
......................................................................aagauguCcucaauguaggcu.................. 10    1     seq
......................................................................Uagauguucucaauguaggcu.................. 2     1     seq
......................................................................aagauguuUucaauguaggcu.................. 9     1     seq
......................................................................aagauguucucaauUuaggcu.................. 2     1     seq
......................................................................aagauguucucaauAuaggcu.................. 8     1     seq
......................................................................aagauguucucaauguagCcu.................. 1     1     seq
......................................................................aagauguucucaauguaggGu.................. 6     1     seq
......................................................................aagauguucucaauguaAgcu.................. 11    1     seq
......................................................................aagauguucCcaauguaggcu.................. 11    1     seq
......................................................................aagauguucucaauguUggcu.................. 1     1     seq
......................................................................aagauguucucGauguaggcu.................. 15    1     seq
......................................................................aagauguucucaauguaCgcu.................. 2     1     seq
......................................................................aagNuguucucaauguaggcu.................. 1     1     seq
......................................................................aagUuguucucaauguaggcu.................. 7     1     seq
......................................................................aagauguucucaauguagUcu.................. 3     1     seq
......................................................................aagauguucAcaauguaggcu.................. 22    1     seq
......................................................................aagaGguucucaauguaggcu.................. 3     1     seq
......................................................................aagauguucucaauguaggcG.................. 206   1     seq
......................................................................aagaAguucucaauguaggcu.................. 37    1     seq
......................................................................aagauguucuAaauguaggcu.................. 22    1     seq
......................................................................aGgauguucucaauguaggcu.................. 5     1     seq
......................................................................aagauguuNucaauguaggcu.................. 1     1     seq
......................................................................aagauUuucucaauguaggcu.................. 2     1     seq
......................................................................aagauguucucaUuguaggcu.................. 5     1     seq
......................................................................aagaCguucucaauguaggcu.................. 10    1     seq
......................................................................aagauguucucaauguaggcC.................. 7     1     seq
```

Star Mature

```
gaucaccauggcgacggaccuugaccgauuugaguaccuauauugcgaaucaucuauccuuucaaaucggaagauguucucaauguaggcuucuuaagucgcgaucaau
......................................................................aNgauguucucaauguaggcu..................	1	1	seq
......................................................................aagauguucuUaauguaggcu..................	38	1	seq
......................................................................aagauguucGcaauguaggcu..................	5	1	seq
......................................................................aagauguucucaauguaggcu..................	56485	0	seq
......................................................................Nagauguucucaauguaggcu..................	14	1	seq
......................................................................aagauguucucaauguagAcu..................	5	1	seq
......................................................................aUgauguucucaauguaggcu..................	8	1	seq
......................................................................aagaugCucucaauguaggcu..................	6	1	seq
......................................................................aagauguucucaaugAaggcu..................	32	1	seq
......................................................................aagauguucucaGuguaggcu..................	8	1	seq
......................................................................aagauguucucaaGguaggcu..................	1	1	seq
......................................................................aagauguucucaauCuaggcu..................	5	1	seq
......................................................................aagauguucucaaugCaggcu..................	4	1	seq
......................................................................aagauguucucaauguagAcuu.................	4	1	seq
......................................................................aagauguCcucaauguaggcuu.................	10	1	seq
......................................................................aagauguucucaauguaggcNu.................	2	1	seq
......................................................................aagauguucucaGuguaggcuu.................	12	1	seq
......................................................................aagauguucucaauguaUgcuu.................	6	1	seq
......................................................................aagauguucGcaauguaggcuu.................	7	1	seq
......................................................................aagauguucucaauguaggNuu.................	1	1	seq
......................................................................aagauguAcucaauguaggcuu.................	36	1	seq
......................................................................aagauguucucUauguaggcuu.................	5	1	seq
......................................................................aagauguucucaauguaggGuu.................	5	1	seq
......................................................................aagauguucuUaauguaggcuu.................	59	1	seq
......................................................................Gagauguucucaauguaggcuu.................	20	1	seq
......................................................................Nagauguucucaauguaggcuu.................	31	1	seq
......................................................................aagauguucucaaugGaggcuu.................	2	1	seq
......................................................................aagaugCucucaauguaggcuu.................	6	1	seq
......................................................................aGgauguucucaauguaggcuu.................	4	1	seq
......................................................................aagauUuucucaauguaggcuu.................	2	1	seq
......................................................................aagauguucucaaCguaggcuu.................	10	1	seq
......................................................................aagUuguucucaauguaggcuu.................	7	1	seq
......................................................................aagauguucucaauguaggcuG.................	45	1	seq
......................................................................aUgauguucucaauguaggcuu.................	5	1	seq
......................................................................aagauguuAucaauguaggcuu.................	14	1	seq
......................................................................aagauguucucaauguaggcGu.................	10	1	seq
......................................................................aaAauguucucaauguaggcuu.................	3	1	seq
......................................................................aagauguucucaauCuaggcuu.................	9	1	seq
......................................................................aagauguucucaaugCaggcuu.................	8	1	seq
......................................................................aagaugGucucaauguaggcuu.................	20	1	seq
......................................................................aagauguucucaauguaAgcuu.................	16	1	seq
......................................................................aagauguucucaaGguaggcuu.................	6	1	seq
......................................................................aagauAuucucaauguaggcuu.................	4	1	seq
......................................................................aagauguucCcaauguaggcuu.................	12	1	seq
......................................................................aagauguucucaauguagUcuu.................	7	1	seq
......................................................................aagauguucucaauAuaggcuu.................	12	1	seq
......................................................................aagauguucucaauguaggAuu.................	38	1	seq
......................................................................aagauguucAcaauguaggcuu.................	35	1	seq
......................................................................aaCauguucucaauguaggcuu.................	2	1	seq
......................................................................aagauguuUucaauguaggcuu.................	12	1	seq
......................................................................aagauguucucaauUuaggcuu.................	1	1	seq
......................................................................aagauguucucaauguaggcCu.................	14	1	seq
......................................................................aNgauguucucaauguaggcuu.................	3	1	seq
......................................................................aagauguucucaauguaCgcuu.................	3	1	seq
......................................................................aagauguucucaaugAaggcuu.................	28	1	seq
......................................................................aagauNuucucaauguaggcuu.................	1	1	seq
......................................................................aagaAguucucaauguaggcuu.................	55	1	seq
......................................................................aagauguucucaauguaggUuu.................	16	1	seq
......................................................................aagaugAucucaauguaggcuu.................	61	1	seq
......................................................................aagaGguucucaauguaggcuu.................	2	1	seq
......................................................................aagaCguucucaauguaggcuu.................	8	1	seq
......................................................................aagauguGcucaauguaggcuu.................	2	1	seq
......................................................................aagauguuGucaauguaggcuu.................	2	1	seq
......................................................................aagauCuucucaauguaggcuu.................	2	1	seq
......................................................................aagauguucucaauguaggcAu.................	24	1	seq
......................................................................aagauguucuGaauguaggcuu.................	2	1	seq
......................................................................aagGuguucucaauguaggcuu.................	5	1	seq
......................................................................aagauguucucaauguaggcuC.................	27	1	seq
......................................................................aagauguucucaUuguaggcuu.................	11	1	seq
......................................................................aagauguucucaaAguaggcuu.................	13	1	seq
```

Star **Mature**

gaucaccauggcgacggaccuugaccgauuugaguaccuauauugcgaaucaucuauccuuucaaaucggaagauguucucaauguaggcuucuuaagucgcgaucaau			
....aagauguucucaauguUggcuu.................	6	1	seq
....aagauguucucGauguaggcuu.................	18	1	seq
....aagauguucucaauguaggcuA.................	31	1	seq
....aagauguucucaauguaggcuu.................	78936	0	seq
....Uagauguucucaauguaggcuu.................	5	1	seq
....aagauguucuAaauguaggcuu.................	20	1	seq
....aagauguucucaCuguaggcuu.................	1	1	seq
....aagauguucucaauguGggcuu.................	10	1	seq
....aagauguucucCauguaggcuuc................	7	1	seq
....aGgauguucucaauguaggcuuc................	122	1	seq
....aagauguucucaauguaggAuuc................	478	1	seq
....Gagauguucucaauguaggcuuc................	282	1	seq
....aagauguucuAaauguaggcuuc................	303	1	seq
....aagauguucucaCuguaggcuuc................	5	1	seq
....aagaugAucucaauguaggcuuc................	904	1	seq
....aagauguucucaauguaggcCuc................	122	1	seq
....aagaGguucucaauguaggcuuc................	60	1	seq
....aagauguucucaauguNggcuuc................	1	1	seq
....aagauguucucaauguUggcuuc................	59	1	seq
....aagauguucucaauguaggcuuN................	10	1	seq
....aUgauguucucaauguaggcuuc................	75	1	seq
....aagauguucucaauguagCcuuc................	36	1	seq
....aagauguucucaauguCggcuuc................	8	1	seq
....aagauguucucaauguaggcNuc................	2	1	seq
....aagauguuAucaauguaggcuuc................	141	1	seq
....aagauguucucaauCuaggcuuc................	80	1	seq
....aagauguucucaauguaggcuuG................	29	1	seq
....aagauguucuNaauguaggcuuc................	1	1	seq
....aagauguucuUaauguaggcuuc................	716	1	seq
....aagauguuUucaauguaggcuuc................	177	1	seq
....aagauguucucaauguaggGuuc................	66	1	seq
....aagauguucucaauguaggcAuc................	261	1	seq
....aagauguCcucaauguaggcuuc................	132	1	seq
....Nagauguucucaauguaggcuuc................	300	1	seq
....aagauguuGucaauguaggcuuc................	76	1	seq
....aagauguucucaauguGggcuuc................	139	1	seq
....aagauguucucaaCguaggcuuc................	107	1	seq
....aagauguucucaauguaggcuuA...............	688	1	seq
....Uagauguucucaauguaggcuuc................	68	1	seq
....aNgauguucucaauguaggcuuc................	36	1	seq
....aagaugGucucaauguaggcuuc................	155	1	seq
....aagauguucucaauguaggUuuc................	104	1	seq
....aagauguucucaUuguaggcuuc................	87	1	seq
....aagauguucucaauguaUgcuuc................	49	1	seq
....aCgauguucucaauguaggcuuc................	3	1	seq
....aagauguucucaauguaggcuAc................	291	1	seq
....aagauguucCcaauguaggcuuc................	127	1	seq
....aagauguucucaaugGaggcuuc................	40	1	seq
....aagauguucucaauguaCgcuuc................	35	1	seq
....aagauguucucGauguaggcuuc................	204	1	seq
....aagauguucucaauguaggcuGc................	28	1	seq
....Cagauguucucaauguaggcuuc................	6	1	seq
....aagauguucucaauguaggcuuc................	962744	0	seq
....aagauguucAcaauguaggcuuc................	386	1	seq
....aagauguucucaaGguaggcuuc................	117	1	seq
....aagauguucucaaugCaggcuuc................	128	1	seq
....aagauguucGcaauguaggcuuc................	56	1	seq
....aaNauguucucaauguaggcuuc................	3	1	seq
....aagauUuucucaauguaggcuuc................	32	1	seq
....aagaAguucucaauguaggcuuc................	558	1	seq
....aagauguucucaGuguaggcuuc................	169	1	seq
....aagauguucucaauUuaggcuuc................	51	1	seq
....aagCuguucucaauguaggcuuc................	8	1	seq
....aagauguGcucaauguaggcuuc................	39	1	seq
....aagauguucucaauguaggcGuc................	81	1	seq
....aagauguAcucaauguaggcuuc................	284	1	seq
....aagauguucucaauguagAcuuc................	123	1	seq
....aaAauguucucaauguaggcuuc................	100	1	seq
....aagauguucucaauAuaggcuuc................	157	1	seq
....aaCauguucucaauguaggcuuc................	28	1	seq

Star **Mature**

gaucaccauggcgacggaccuugaccgauuugaguaccuauauugcgaaucaucuauccuuucaaaucggaagauguucucaauguaggcuucuuaagucgcgaucaau			
....aaUauguucucaauguaggcuuc................	63	1	seq
....aagauguucucaaAguaggcuuc................	175	1	seq
....aagaugCucucaauguaggcuuc................	120	1	seq
....aagaCguucucaauguaggcuuc................	107	1	seq
....aagauguucucaauguaggcuCc................	151	1	seq
....aagauguucucUauguaggcuuc................	40	1	seq
....aagUuguucucaauguaggcuuc................	76	1	seq
....aagGuguucucaauguaggcuuc................	110	1	seq
....aagauguucuGaauguaggcuuc................	98	1	seq
....aagauguucucaauguaggcuuU................	4386	1	seq
....aagauCuucucaauguaggcuuc................	69	1	seq
....aagauguucucaauguagUcuuc................	71	1	seq
....aagauNuucucaauguaggcuuc................	6	1	seq
....aagauAuucucaauguaggcuuc................	113	1	seq
....aagauguucucaauguaAgcuuc................	219	1	seq
....aagauguucucaaugAaggcuuc................	504	1	seq
....aagauguucNcaauguaggcuuc................	1	1	seq
....aagauguucucaauguaggcuucu...............	4052	0	seq
....aagauguucucaauguaggcuuAu...............	7	1	seq
....aagaAguucucaauguaggcuucu...............	2	1	seq
....aagauguucucaauguaggcuuGu...............	2	1	seq
....aagaugGucucaauguaggcuucu...............	1	1	seq
....aagauguucuUaauguaggcuucu...............	3	1	seq
....aagauguucucGauguaggcuucu...............	1	1	seq
....aagaugAucucaauguaggcuucu...............	3	1	seq
....aagauguucucaauguaAgcuucu...............	1	1	seq
....aagauguucucaaAguaggcuucu...............	1	1	seq
....aagauguucucaauguaggcCucu...............	1	1	seq
....aagauguucAcaauguaggcuucu...............	2	1	seq
....aagauguAcucaauguaggcuucu...............	1	1	seq
....aagauguucucaauguaggAuucu...............	1	1	seq
....aagauguucCcaauguaggcuucu...............	1	1	seq
....aagauguucucaauguaggcuucG...............	2	1	seq
....aagaugCucucaauguaggcuucu...............	1	1	seq
....aagauguucucaauguaggcuuUu...............	120	1	seq
....aagauguuGucaauguaggcuucu...............	1	1	seq
....aagauguucucaaCguaggcuucu...............	1	1	seq
....aagauguucucaaugAaggcuucu...............	3	1	seq
....aagauguucucaauguaggcuAcu...............	1	1	seq
....aagauguucucaauguaggcuucN...............	2	1	seq
....aagauguucucaauguaggcuucA...............	98	1	seq
....aagauguucucaauguagAcuucu...............	1	1	seq
....aagauguucucaauAuaggcuucu...............	1	1	seq
....aagauguucucaauguaggcuuUuu..............	9	1	seq
....aagauguucucaauguaggcuucAu..............	8	1	seq
....aagauguucucaauguaggcuucuu..............	34	0	seq
....agauguucucaauguaggcu..................	34	0	seq
....agauguucucaauguaggcuu.................	27	0	seq
....agauguucucaaugAaggcuuc................	1	1	seq
....agauguAcucaauguaggcuuc................	2	1	seq
....agauguucucaUuguaggcuuc................	1	1	seq
....agaugAucucaauguaggcuuc................	1	1	seq
....agauguucucaauguaggcuuc................	639	0	seq
....agauguucucaauguaggcuuU................	3	1	seq
....agauguucucaaAguaggcuuc................	1	1	seq
....agauguucuUaauguaggcuuc................	1	1	seq
....agauguucucaauAuaggcuuc................	1	1	seq
....agauguucucaauguaggcuucu...............	7	0	seq
....gauguucucaauguaggcu..................	4	0	seq
....Aauguucucaauguaggcuu.................	1	1	seq
....gauguucucaauguaggcuuc................	16	0	seq
....Uauguucucaauguaggcuuc................	1	1	seq
....auguucucaauguaggcu..................	2	0	seq
....auguucucaauguaggcuu.................	2	0	seq
....auguucucaauguaggcuuc................	7	0	seq
....aAguucucaauguaggcuuc................	1	1	seq
....uguucucaauguaggcuu.................	3	0	seq
....ugAucucaauguaggcuuc................	1	1	seq
....uguucucaauguaggcuuc................	68	0	seq
....guucucaauguaggcuuc................	8	0	seq

Star Mature

gaucaccauggcgacggaccuugaccgauuugaguaccuauauugcgaaucaucuauccuuucaaaucggaagauguucucaauguaggcuucuuaagucgcgaucaau
